# Decision maker views on priority setting in the Vancouver Island Health Authority

**DOI:** 10.1186/1478-7547-6-13

**Published:** 2008-07-21

**Authors:** Francois Dionne, Craig Mitton, Neale Smith, Cam Donaldson

**Affiliations:** 1 Department of Health Care and Epidemiology, University of British Columbia, 5804 Fairview Avenue, Vancouver, BC, V6T 1Z3, Canada; 2 Health Studies, University of British Columbia Okanagan, 3333 University Way, Kelowna, BC, V1V 1V7, Canada; 3 Child and Family Research Institute, 950 West 28th Avenue, Vancouver, BC, V5Z 4H4, Canada; 4 Institute of Health and Society, Newcastle University, 21 Claremont Place, Newcastle upon Tyne, NE2 4AA, UK; 5 Health Studies, Faculty of Health and Social Development, University of British Columbia Okanagan, 3333 University Way, Kelowna, BC, V1V 1V7, Canada

## Abstract

**Background:**

Decisions regarding the allocation of available resources are a source of growing dissatisfaction for healthcare decision-makers. This dissatisfaction has led to increased interest in research on evidence-based resource allocation processes. An emerging area of interest has been the empirical analysis of the characteristics of existing and desired priority setting processes from the perspective of decision-makers.

**Methods:**

We conducted in-depth, face-to-face interviews with 18 senior managers and medical directors with the Vancouver Island Health Authority, an integrated health care provider in British Columbia responsible for a population of approximately 730,000. Interviews were transcribed and content-analyzed, and major themes and sub-themes were identified and reported.

**Results:**

Respondents identified nine key features of a desirable priority setting process: inclusion of baseline assessment, use of best evidence, clarity, consistency, clear and measurable criteria, dissemination of information, fair representation, alignment with the strategic direction and evaluation of results. Existing priority setting processes were found to be lacking on most of these desired features. In addition, respondents identified and explicated several factors that influence resource allocation, including political considerations and organizational culture and capacity.

**Conclusion:**

This study makes a contribution to a growing body of knowledge which provides the type of contextual evidence that is required if priority setting processes are to be used successfully by health care decision-makers.

## Background

Despite the fact that most hospital and physician services are publicly funded in Canada (Canadian Medicare covers about 98% of hospital and physician costs), there are limits to the resources available to pay for these services. Further, given that there are very few constraints on the growth of demand for these services [1], it is not surprising to find that, in a context where governments are focused on cutting taxes, decisions regarding the allocation of the available resources are a subject of growing conflict, and a growing source of dissatisfaction for decision-makers [2].

In most health care organizations, resource allocation decisions are typically based on historical spending patterns, adjusted through targeted budget increases related to political and demographic influences [2]. This means that gaps in service availability can only be addressed through increases in the organization's funding or through cost minimization strategies (to free up some money). Dissatisfaction with the results of historical allocation patterns have led to an increased interest in research on more explicit, evidence-based resource allocation processes in health care [3]. An emerging area within this research has been the empirical analysis of the characteristics of existing and desired priority setting processes, as well as the structural features of health care organizations that hinder the implementation of desired processes [4], from the perspective of decision-makers. The goal is to describe what health care decision-makers want in a priority setting process and what they see as barriers to implementing such processes.

This paper presents information obtained through interviews of decision makers in the Vancouver Island Health Authority (VIHA), one of six health authorities in British Columbia, Canada. This study was the first step in a research project aimed at transforming the priority setting practices within VIHA towards a more formal, evidence-based process (known as program budgeting and marginal analysis, or PBMA).

The primary objectives of this study were to develop an understanding of the characteristics of historical resource allocation practices, to determine what institutional features shaped these practices and to identify desired improvements from the perspective of decision-makers in an organization committed to the implementation of a formal priority setting process. Specifically, this study asked decision-makers in a regional health authority to describe the features of their ideal priority setting process and to assess current practices against this standard. The orientation of this paper is towards the operationalization of a formal priority setting framework, not merely justification for implementation of such a framework.

There is a growing body of knowledge on decision-makers' perspective on priority setting and resource allocation processes but it includes very limited information from decision-makers in integrated health care organizations where a formal priority setting process is actually being implemented. Greener and Powell [5], for example, surveyed senior decision-makers in the 121 health authorities in England and Wales to examine approaches to priority setting and resource allocation. Some of the respondents used formal priority setting processes while others did not, but results are not differentiated between those two groups making it impossible to measure the association between the use of formal priority setting processes and satisfaction with resource allocation decisions made. Their overall conclusion was that, despite an explicit desire from Government to have health authorities adopt an evidence-based resource allocation process, very slow progress has been made in that direction. The two main reasons cited for this are: 1) cynicism on the part of the health authorities with constantly changing Government plans; and 2) a path-dependent budget making process (a process that reinforces historical patterns) which only permits changes at the margins.

Mitton and Prout [6] surveyed decision-makers of a regional health care organization in Australia that was considering implementing a formal priority setting process. They found strong support for moving toward such a process. The main desired features of the process were a commitment from the Government to follow-through with full implementation and acceptance of the results, and means to improve intra-organizational coordination. Challenges identified included concerns over the system-wide impact of a priority setting process, particularly in terms of its effect on small towns, political interference, and organizational dynamics (e.g., level of trust within the organization). This health care organization did not adopt a formal priority setting process.

Martin et al [7] interviewed members of two committees charged with priority setting for disease-specific new technologies in Ontario, and focused on the perceived fairness of their processes. They found the extent to which stakeholders' perspectives are included in the process to be a key determinant of perceived fairness. Most respondents stated that fairness depends on the inclusion of the perspectives of all parties affected and in a way that is honest and understandable by all. Other determinants of fairness were identified as consensus decision-making and transparency of the process.

Jan [8] approached the question of what decision-makers want in a priority setting process from a theoretical perspective by discussing the impact of institutional context on the success of a priority setting process. His primary assertion was that a typical priority setting framework relies heavily on "the goodwill of participants in providing realistic assessment of expected benefits" [[8], p.633] in order to collectively achieve "efficiency gains" (p.634). Attaining the goodwill required to achieve collective gains depends on the strength of the link between collective gains and individual interest. The weaker this link, the greater the incentive for 'gaming' the process, which, added to the incomplete information available on benefits and costs, leads to significant limitations to the potential benefits of priority setting processes. Jan proposes three main solutions: 1) increasing the information on program costs and benefits in the organization; 2) limiting the number of alternatives considered in a priority setting exercise and 3) ensuring long-term commitment to the organization from the decision-makers (through contracts) so that they see their forecasts through.

It is clear that a thorough understanding of current priority setting/resource allocation practices, what shapes these practices, and what decision-makers see as key areas for improvement, are essential pieces of information in the development of a well-designed resource allocation process. Such information can also provide a roadmap to this process where anticipated barriers are identified. It is also clear that a study of decision-makers in an integrated health care organization that is implementing a formal priority setting process will fill a gap in the spectrum of existing studies.

## Methods

### Context

The Vancouver Island Health Authority (VIHA) is responsible for the provision of health care services to a population of about 730,000 people in a mix of urban and rural environments. This health authority has approximately 16,000 employees, operates 15 acute care hospitals, is served by about 1,600 physicians, and has an annual operating budget of $1.4 billion CAN (2007). At the time of the interviews (Fall 2005), and continuing since then, VIHA has been involved in an organizational re-structuring with the objective of creating an integrated organization providing services across the full continuum of care. Key features of the new organizational model are co-management of clinical portfolios (administrative and medical directors) and devolution of decision-making closer to the front line (i.e., matching authority to responsibility). The re-design of the priority setting practices was seen by the CEO and the 10 member Executive team as part of this organizational re-structuring.

### Design and analysis

In-depth, face-to-face interviews were conducted with 18 senior managers and medical directors within VIHA in the Fall 2005 (the questionnaire is attached as Appendix A). Respondents were purposively selected to achieve a heterogeneous sample, including a breadth of priority setting experience and roles in the health authority [9]. Approximately one quarter of respondents were physicians while the others were professional managers/administrators, although some of those would have a clinical background. The questionnaire was developed based on previous experience elsewhere [6] and was further informed through an updated review of the literature. Some of the questions were open-ended while others asked for the respondents' perception in relation to a set of specific process evaluation criteria such as: fairness, information dissemination, use of research evidence, appeal process, and stakeholder representation.

Interviews were recorded and then transcribed. A research team member analyzed the contents of the transcripts using the N*6 qualitative analysis software package. Major themes and sub-themes were developed until theoretical saturation was reached and no new themes were identified [10,11]. The code structure was refined until the themes, or categories of meaning, had internal convergence and external divergence (i.e., the categories were internally consistent but distinct from one another) [12]. A second research team member independently coded a sample of the transcripts to ensure that consistent patterns of information emerged. The study was approved by the Behavioral Research Ethics Board at the University of British Columbia.

## Results

This section focuses on two main areas of findings from the interviews. First, we indicate the characteristics which respondents identify as desirable in a resource allocation process – identified either directly or by comparison with their experience in previous priority setting efforts. Second, we describe a number of factors, identified by the respondents, which determine or shape the prospects for formalized resource allocation activity.

### Characteristics of existing and desired resource allocation processes

Through the interviews a set of nine features describing the desired resource allocation process at VIHA emerged. In this sub-section, we define these features and use them as criteria against which the past priority setting practices can be evaluated.

The first desired feature is *baseline assessment*, or the inclusion in the priority setting process of existing activities so that an appropriate level of funding for these activities can be determined. Overall, respondents felt that *baseline assessment *was lacking in past priority setting processes: "we assume when a new program comes into play... that the baseline is correct. And I think there should be a review on the front end to ascertain whether the baselines are in fact correct. And I think that that's a gap in this process".

The second feature is the *use of best evidence *embedded in the workings of the process. There are currently mixed opinions as to whether the past processes delivered on this criterion. For example, one respondent stated, "Yeah, I would think we've tried to be evidence-based as much as possible", while another argued that "I think it's been haphazard and ad hoc".

The third feature is *clarity*, meaning a process that is clear, explicit and transparent. The respondents suggested that past processes failed on this criterion, although some respondents expressed the view that the potential for clarity exists. Responses ranged from: "I would say the actual process or processes are probably, generally speaking, not too explicit" and " There have been things approved and we've heard about it through the grapevine and it hasn't been transparent" to "Fairly constant methodology used actually... very, very focused and clear leadership-that's fundamental, right? So to me it looks like the process has potential".

The fourth feature is *consistency *referring to a process that is applied uniformly across the organization and survives over time. The processes employed prior to Fall 2005 were judged to be lacking on this criterion: "We've had multiple processes, multiple criteria, multiple rationales and changes in decision makers over the last five to ten years" and "It hasn't been consistent... you do seem to have these double standards".

The fifth desired feature is *quality criteria*, defined as decision criteria that are clear, measurable and relevant to the organization. Consensus opinion on the performance of past processes in regard to this criterion was negative. Criteria were found to be lacking clarity, ability to discern between proposals and consistency. For example, one respondent stated: "The evidence was always there... but there was no criteria to say whose (department) was the most needy".

*Dissemination *is the sixth feature. It refers to the built-in communication and explanation, throughout the organization, of all aspects of the process, including decision criteria, actual decisions and rationales. Performance of past processes in terms of this criterion was rated as mixed in relation to internal stakeholders and lacking with respect to external stakeholders. With internal stakeholders, communication efforts were found to be insufficient by many respondents while some judged these efforts to be sufficient. Opinions ranged from: (in assessing communication efforts) "I don't think we have in the past done well at that and even last year. I don't think we did as well as we could have" and " Communicating with our care providers and our middle management and our staff about why certain decisions around priorities have been made probably hasn't occurred at a detailed level very well" to (in answering the same question) "I would have to say... that the answer is yes... they do a very good job of telling us what we hope to do, why they made the decisions that they made and what to do if you felt that there was a need to respond to an appeal around that".

The seventh feature is *evaluation*; the process should have a built-in evaluation component that would ensure ongoing documentation of the activities and assessment of the impact of the resulting budget decisions. This feature did not exist in priority setting processes prior to the Fall 2005.

The eight feature of desired priority setting processes is appropriate stakeholder *representation*. Just like dissemination, *representation *is broken down into internal and external stakeholders. On both fronts, opinions were mixed on the performance of past processes. With regard to internal stakeholders: "It seems to that what I've seen most recently in the organization is (more of a collaborative process at the middle management level) with some input from providers or from people who are close to the action within each programs...and then of course, a lens applied by more senior people to that prioritization" and "it just didn't lead to a feeling that people had had input and an opportunity to advocate for what they thought was important perhaps as well as it could have". As for external stakeholders, i.e. the public: "I do know that the... public input is brought to processes or brought to decisions that come from the program areas, so wherever there are Advisory Committees, or Councils, or whatever within the program, that information does help to inform the program, where they get their priorities" and "I can't recall off the top of my head any specific examples of the public being actively involved in any priority setting."

Finally, the ninth feature is a link to the *strategic direction *of the organization. The priority setting process should clearly reflect, in all its operations, the strategic direction established for the organization. According to those interviewed, this linkage was limited in past processes.

### Determinants and challenges

Respondents identified several factors that influence or determine the shape of the resource allocation process, i.e. factors that can help explain the divergence between existing and desired processes. These factors can be classified under two main themes: political considerations and organizational culture and capacity.

Respondents thought that political forces often directly shaped the allocation decisions. The most important of these political forces was seen to be the provincial Ministry of Health. "Health care is a huge political issue and the reality of that is that governments who fund the health authorities get caught up in the decisions of the health authorities and it becomes political" – overriding other factors that might be considered during formal priority setting activities.

Political decisions have also resulted in repeated and extensive restructuring of VIHA in recent years. This organizational change has, at a minimum, hampered the development of a stable system of priority setting. This has affected negatively VIHA's performance in areas such as the consistency of resource allocation choices through time, across departments and among different stakeholders, and the dissemination of information about the process and the decision criteria. The instability has also held back efforts to create shared vision, goals, and strategic directions on an organization-wide basis.

Also, political decisions, related primarily to a focus on tax cuts, have made resources very tight. An environment of fiscal constraint has enveloped VIHA since its establishment. This has shaped the organization's culture and has been internalized by the decision makers. It is reflected in a lack of interest by some in formal mechanisms for prioritization; according to one respondent, "we didn't need a formalized process for investing a lot of money because we didn't have a lot of money to invest". In VIHA, according to another, "we come from a scarcity mentality... where you protect your resources... you don't share those resources. And I think that's a challenge".

The other category of determinants and challenges is the organizational culture and capacity. One important way the organizational culture affects the priority setting process is through the development of a shared vision throughout the organization. Resource allocation in an integrated health system like VIHA can occur *within *portfolios (defined as a group of related programs, for example diagnostic and surgical services) or *across *portfolios; that is, the scope of prioritization can be relatively narrow or more broadly defined. Many felt the latter was most desirable: "isn't a bed replacement plan equally important as diagnostic equipment which is just as important as some of the other things"? However, to carry out reallocations across portfolios, values related to different parts of the organization, providing different types of services, must be ranked so that the relative merit of any given proposal can be assessed. "One of the complexities of life in health authorities is the relationship between life and death services and residential services and palliative services and prevention services". Most of the respondents thought that the values from the different parts of VIHA have not been integrated into a cohesive shared vision that would support such an undertaking. This integration was seen as likely to be a difficult task: "Care and compassion, client-focus, healthy workplace... all those kinds of things are not always front and center on that priority setting agenda. I would like to see them articulated more clearly, maybe more measurably."

The scarcity mentality, the lack of experience working together, and the lack of shared vision may all contribute to the fears expressed by some respondents that it might prove impossible to establish a fair priority setting process across the portfolio boundaries of VIHA: "life-saving priorities would always be ranked higher than rehabilitation priorities".

Finally, respondents expressed concerns over the organizational capacity in terms of time and skills required to implement a resource allocation process and operationalize it: "it's not that there isn't a lot of motivation to do evidence-based policy or budgeting decisions but the capacity is limited around the resource and skills and time and the tools that the decision-makers have to have to do that". Organizational capacity as it relates to the information requirements of a priority setting process is another challenge: "I think a large barrier to allocating resources whether it was in the past or now is good information, is having really good systems that allow us to get information that truly can inform us".

## Discussion

Under the leadership of senior management, VIHA has undergone a fundamental restructuring over the last three years. One of the areas specifically addressed in this re-structuring is the priority setting/resource allocation process. In our interviews, we asked decision makers at VIHA to reflect upon their previous approaches to priority setting and to identify features that would characterize an improved or ideal model. Our purpose was to explore how decision-makers assess past priority setting processes by comparing them to their self-described ideal process. This investigation has produced information on those areas of priority setting processes where the greatest need for/prospect of improvements exist, and therefore on the criteria against which the value of any new process is most likely to be judged. We also uncovered a range of determinants and challenges that will influence an organization's ability to move toward this desired future.

This information has implications for both researchers and decision-makers. For researchers, it provides direction for future refinements to priority setting implementation procedures. For decision-makers, it presents a checklist against which current practices can be assessed and shortcomings identified.

Several features of priority setting processes that emerged from our interviews are in line with previous research findings. This was due in part to the fact that respondents were probed on features that we specifically extracted from the literature (e.g. features related to ethical considerations, such as those contained in the Accountability for Reasonableness framework [13]). Our paper builds on previous work in Canada and confirms previous findings. For example, Mitton and Donaldson [14] listed a number of desired features of priority setting processes including: physician buy-in, transparency, stakeholder engagement, strategic links, and greater accountability. All of these were highlighted in our study. Similarly, Teng et al. [15] also listed desired improvements in priority setting such as: transparency, defensibility, consistency and fairness.

However, the current paper goes further in defining the desired characteristics of priority setting processes. For instance, defining goals and outcomes for the process had been identified as desirable in both previous studies in Western Canada. Our study provides further clarity regarding the nature of those goals, specifically a desire to use priority setting processes to review baseline spending i.e. not just to guide new spending. Another example is the issue of decision criteria. Elsewhere decision-makers discussed a process that is explicit, that is linked to strategic direction and that is transparent. Our current work has linked these characteristics directly to the decision criteria that are used in the process. Here we found that decision-makers need to define criteria that are clear and measurable. Implications of this are that: 1) implementation procedures should include a more detailed definition of the characteristics of decision criteria to be used; and 2) when decision makers assess their current practices, their review of decision criteria should go beyond the fit with strategic directions.

In terms of international comparisons, determinants and challenges to the priority setting process identified by respondents in VIHA are in line with what was described by Greener and Powell [5] based on work in the UK. Similarly, in work from Australia, Mitton and Prout [6] refer specifically to the influence of political considerations on priority setting processes. Furthermore, organizational capacity and culture was raised by Jan [8] as a critical determinant of the success of a priority setting process. Our study provides further illustrations of how these determinants and challenges can manifest themselves in the implementation of a formal priority setting process in an integrated health care organization.

Finally, our findings support those of Bate et al [16] who examined how prioritization decisions are understood and managed by decision-makers in the National Health Service (NHS) in England. Their conclusion was that "Commissioning as undertaken in practice, deviates from what can be surmised from the guiding principles initially outlined by decision-makers and consequently performs poorly in relation to these" [[17], p.10]. In other words, decision-makers in England, just as on Vancouver Island, know what they would like to do in terms of priority setting but in practice are far from their goal. Not surprisingly, this results in decisions that are not satisfying to them.

The main limitation of the current study is the fact that respondents were aware that these interviews were to provide a baseline in a project that introduces a new priority setting process. Knowing that the Executive team had already decided to change the existing process as part of the corporate restructuring might have influenced the responses; on the one hand, some respondents might be looking for ways to justify the decision to make the change while on the other hand some might feel more free to be honest given that they would not be stuck with a process they criticized. It is difficult to know which of these influences is present, and to what extent. Furthermore, as data collection and data analysis did not take place concurrently, it was not possible to refine the interview guide in response to data as the study progressed.

## Conclusion

As the focus on resource allocation decisions in healthcare sharpens, the dissatisfaction of decision-makers with prevailing priority setting processes, mostly based on historical patterns, is rising. In response, research on alternatives to existing processes is gathering increasing interest. For this research to provide workable solutions, it needs to be contextualized, as Lomas et al explain [[17], p.3]: "evidence has little meaning or importance for decision-making unless it is adapted to the circumstances of its application. ... Scientific evidence on what works should be combined with scientific evidence on context."

In this study, we have summarized the views of decision-makers at VIHA regarding their past experience with and their hopes for priority setting processes. To date, little research on the perspectives of decision-makers in integrated health care organizations on priority setting frameworks has been done. This study makes a contribution to the growing body of knowledge on decision-makers' perspective on priority setting processes which is the type of contextual evidence that is required if these processes are to be used successfully by health care decision-makers.

Our findings confirm that decision-makers understand the value of formal priority setting processes and a clear description of what they would like such processes to look like is emerging. The next step is implementation of this knowledge, which will require explicit handling of the identified challenges. The fact that this knowledge is grounded in the reality of the decision-makers' everyday life provides a solid base to work from.

## Competing interests

The authors declare that they have no competing interests.

## Authors' contributions

FD drafted the manuscript. CM advised on the interview plan, including formulation of the questionnaire, provided direction for the drafting of the manuscript and suggested revisions to the manuscript. NS assisted with the thematic analysis of the interviews and contributed to the drafting of the manuscript. CD provided significant comments on the content and the organization of the manuscript. All authors read and approved the final manuscript.

## Appendix A

Questions for one-on-one interviews with Vancouver Island Health Authority decision-makers on past, present and future priority setting processes

1 Can you please describe the process or processes that have been used in the past to identify priorities and allocate resources across major program areas within the Vancouver Island Health Authority (VIHA)?

2 Overall, do you think the process or processes employed in the past have worked well? How would you define 'success' in this instance?

3 What specific barriers have been faced in the past when setting priorities and allocating resources?

4 Overall, how fair do you think the process (or processes) have been?

4a How well have the process, decision criteria, and rationale on which decisions have been based been disseminated within or outside the organization?

4b In your view, have decisions been made that are based on the best available evidence, and in essence would be deemed to be 'reasonable' by fair minded parties?

4c Has there been an explicit process for appealing resource allocation decisions once made?

4d To your knowledge, has the organization dedicated resources to ensuring that the process and decisions are adequately communicated, that the decisions are based on reasonable evidence and that an appeals process has been developed?

5 How could the past processes of setting priorities and allocating resources be improved? Please be as specific as possible

6 What factors do you think are necessary for sustaining an explicit, formal, priority setting process in VIHA? Please be as specific as possible.

7 How has the public been used in priority setting/resource allocation processes in the past? How would you want the public to be involved in the priority setting process?

8 What role have physicians played in priority setting/resource allocation processes in the past? How would you want the physicians to be involved in the priority setting process?

9 How well do you think the values of VIHA have been incorporated into priority setting activity? How should the values of VIHA be incorporated into the priority setting process?
